# Relationship Between DTI Metrics and Cognitive Function in Alzheimer’s Disease

**DOI:** 10.3389/fnagi.2018.00436

**Published:** 2019-01-09

**Authors:** Chantel D. Mayo, Mauricio A. Garcia-Barrera, Erin L. Mazerolle, Lesley J. Ritchie, John D. Fisk, Jodie R. Gawryluk, Alzheimer’s Disease Neuroimaging Initiative

**Affiliations:** ^1^Department of Psychology, University of Victoria, Victoria, BC, Canada; ^2^Department of Radiology, Hotchkiss Brain Institute, University of Calgary, Calgary, AB, Canada; ^3^Department of Clinical Health Psychology, University of Manitoba, Winnipeg, MB, Canada; ^4^Department of Psychology, Nova Scotia Health Authority, Halifax, NS, Canada; ^5^Department of Psychiatry, Psychology and Neuroscience, Dalhousie University, Halifax, NS, Canada; ^6^Department of Medicine, Dalhousie University, Halifax, NS, Canada

**Keywords:** Alzheimer’s disease, magnetic resonance imaging, diffusion tensor imaging, white matter, memory, executive function

## Abstract

**Introduction:** Alzheimer’s disease (AD) is a neurodegenerative disorder with a clinical presentation characterized by memory impairment and executive dysfunction. Our group previously demonstrated significant alterations in white matter microstructural metrics in AD compared to healthy older adults. We aimed to further investigate the relationship between white matter microstructure in AD and cognitive function, including memory and executive function.

**Methods:** Diffusion tensor imaging (DTI) and neuropsychological data were downloaded from the AD Neuroimaging Initiative database for 49 individuals with AD and 48 matched healthy older adults. The relationship between whole-brain fractional anisotropy (FA), mean diffusivity (MD), axial diffusivity (AxD), radial diffusivity (RD), and composite scores of memory and executive function was examined. We also considered voxel-wise relationships using Tract-Based Spatial Statistics.

**Results:** As expected, individuals with AD had lower composite scores on tests of memory and executive function, as well as disrupted white matter integrity (low FA, high MD, AxD, and RD) relative to healthy older adults in widespread regions, including the hippocampus. When the AD and healthy older adult groups were combined, we found significant relationships between DTI metrics (FA/MD/AxD/RD) and memory scores across widespread regions of the brain, including the medial temporal regions. We also found significant relationships between DTI metrics (FA/MD/AxD/RD) and executive function in widespread regions, including the frontal areas in the combined group. However, when the groups were examined separately, no significant relationships were found between DTI metrics (FA/MD/AxD/RD) and memory performance for either group. Further, we did not find any significant relationships between DTI metrics (FA/MD/AxD/RD) and executive function in the AD group, but we did observe significant relationships between FA/RD, and executive function in healthy older adults.

**Conclusion:** White matter integrity is disrupted in AD. In a mixed sample of AD and healthy elderly persons, associations between measures of white matter microstructure and memory and executive cognitive test performance were evident. However, no significant linear relationship between the degree of white matter disruption and level of cognitive functioning (memory and executive abilities) was found in those with AD. Future longitudinal studies of the relations between DTI metrics and cognitive function in AD are required to determine whether DTI has potential to measure progression of AD and/or treatment efficacy.

## Introduction

Approximately 47.5 million people are living with dementia, the majority of which have Alzheimer’s disease (AD; World Health Organization, 2016). AD is a neurodegenerative disorder typically characterized by memory loss, although other cognitive domains, including executive functions, are often affected as well (Kirova et al., 2015; Alzheimer’s Association, 2017). As there is no cure for AD, early detection of AD pathology is critical to allow for intervention at the earliest possible time point.

Emerging evidence that brain pathology precedes clinical symptoms – up to 10–20 years before the dementia stage – has contributed to a surge of researchers seeking to identify pre-symptomatic change through the use of biomarkers based on a variety of techniques, including magnetic resonance imaging (MRI; Sperling et al., 2011; Bernard et al., 2014; Hampel et al., 2014; Wurtman, 2015). MRI is an ideal tool for detecting early brain changes because it is non-invasive and easily repeatable. To date, the majority of MRI research on AD has focused on neurodegeneration in gray matter structures (Teipel et al., 2013; Cash et al., 2014). These findings indicate widespread, whole brain atrophy in individuals with AD, including enlarged ventricles and decreased hippocampal volume (Perl, 2010; Hampel et al., 2014). However, there is less research on white matter, which is also affected early in the disease process (Sachdev et al., 2013), and the relationship between white matter integrity and cognitive function in AD.

The Amyloid Cascade Hypothesis (Hardy and Higgins, 1992) predicts that axonal degeneration is a result of Wallerian degeneration and precedes neuronal loss. The close association of tau with both axonal integrity and with the cognitive symptoms of AD suggests that white matter changes may occur independently and perhaps prior to changes in gray matter. Furthermore, the retrogenesis model (Bartzokis et al., 2007) suggests that white matter degeneration in AD follows a reverse temporal pattern to that observed in myelination. Together these support the idea that characterizing initial white matter changes may be particularly helpful for early identification of AD.

One promising method of detecting early white matter microstructure changes is an MRI-based technique called diffusion tensor imaging (DTI); DTI is based on water diffusion within the brain (Alexander et al., 2007; Soares et al., 2013). Frequently reported DTI metrics include fractional anisotropy (FA), a measure of the degree of directionality of water diffusion, and mean diffusivity (MD), the mean water diffusion rate (Alexander et al., 2007; Soares et al., 2013; Amlien and Fjell, 2014; Bennett and Madden, 2014; Alves et al., 2015). More recently, reported DTI metrics have also included axial diffusivity (AxD), the rate of water diffusion along the longitudinal axis and radial diffusivity (RD), the rate of water diffusion along the perpendicular axes (Alexander et al., 2007; Alves et al., 2015). Together, FA and MD provide information about changes to barriers to diffusion; increased AxD has been associated with axonal degeneration and increased RD has been linked to demyelination (Bosch et al., 2012; Bennett and Madden, 2014; Kantarci, 2014; Alves et al., 2015).

One of the first DTI meta-analyses of 41 region of interest studies (Sexton et al., 2011) found widespread alterations in FA and MD. Low FA was found in the white matter of the frontal and temporal lobes, the genu and splenium of the corpus callosum, the anterior, middle and posterior cingulum, the parahippocampal cingulum, the uncinate fasciculus, and the superior longitudinal fasciculus. Additionally, high MD was found in the white matter of all the lobes of the brain, the genu and splenium of the corpus callosum, the posterior cingulum, the uncinate fasciculus, and the hippocampus. A more recent meta-analysis of 13 whole-brain TBSS studies also found low FA and high MD in AD when compared to healthy aging adults, generally within the parietal, temporal, and prefrontal white matter, including association fibers and interhemispheric connections via the corpus callosum (Acosta-Cabronero and Nestor, 2014).

Longitudinal within-group studies have also demonstrated white matter changes across time in AD (e.g., Kitamura et al., 2013; Norwrangi et al., 2013; Genc et al., 2016; Elahi et al., 2017). For example, Genc et al. (2016) examined individuals with AD at 6 months relative to baseline and found widespread white matter alterations (decreased FA and increased MD, AxD, RD) in the corpus callosum, superior longitudinal fasciculus, and cingulum bilaterally. Furthermore, Elahi et al. (2017) examined individuals with AD at 1 year follow-up compared to baseline and also found widespread white matter alterations in FA and MD, largely within posterior regions.

Recently, our group demonstrated significant alterations in white matter microstructure metrics in individuals with AD compared to matched healthy older adults (Mayo et al., 2017). Specifically, we found that, relative to healthy older adults, individuals with AD had lower FA and higher MD in the hippocampal cingulum, as well as the corpus callosum, internal and external capsule, corona radiata, posterior thalamic radiation, superior and inferior longitudinal fasciculi, fronto-occipital fasciculus, cingulate gyri, fornix, uncinate fasciculus, and tapetum. These observed alterations in white matter integrity were consistent with previous cross-sectional DTI studies of AD (Stricker et al., 2009; Serra et al., 2010; Douaud et al., 2011; Liu et al., 2011; Shu et al., 2011; Bosch et al., 2012; Lim et al., 2012; Sousa Alves et al., 2012). However, we also found that while both individuals with AD and healthy older adults had reductions in FA and increased MD over a period of 1 year in widespread regions, although the changes were more extensive in AD and more specific to the medial temporal lobe.

Alterations in white matter microstructure are thought to contribute to cognitive decline due to progressive “disconnection” between cortical regions (O’Sullivan et al., 2001). Some investigators have found a relationship between DTI indices and more global measures of cognitive function in individuals with AD (e.g., Bozzali et al., 2002; Lee et al., 2002; Shim et al., 2017). For example, Nir et al. (2013) found that FA, MD, AxD, and RD in the left hippocampal cingulum and left fornix were strongly associated with MMSE scores, while FA, MD, AxD, and RD in the left cingulum, bilateral sagittal stratum, and bilateral inferior fronto-occipital fasciculus were related to Clinical Dementia Rating scores, and FA and MD in the left hippocampal cingulum were related to AD Assessment Scale (ADAS) cognitive scores. More recently, Kantarci et al. (2017) found that lower FA and higher MD in the ventral cingulum and precuneus white matter were associated with lower MMSE and high Clinical Dementia Rating box scores; and higher MD was associated with lower MMSE and higher Clinical Dementia Rating box scores in the entorhinal white matter. In contrast, other studies have not observed any relationship between DTI metrics and MMSE (e.g., Stahl et al., 2007; Ibrahim et al., 2009; Patil and Ramakrishnan, 2014). For example, Patil and Ramakrishnan (2014) did not find any significant relationships between DTI metrics (FA, MD, AxD, RD) and MMSE scores in any explored brain region (left fornix, left splenium of corpus callosum, right splenium of corpus callosum, genu of corpus callosum) in individuals with AD. Such mixed findings likely reflect the use of different methodological and analytic approaches (e.g., whole brain vs. region of interest), selection of different regions of interest, use of different cognitive test batteries, and different group characteristics (e.g., some studies combine individuals with mild cognitive impairment and AD).

Relatively fewer studies have examined the relationship between white matter microstructure and specific measures of memory and executive function in AD. In one study, Mielke et al. (2009) found that lower FA corresponded to poorer performance on tests of memory [California Verbal Learning Test, Wechsler Memory Scale (WMS) Logical Memory] and executive function (Trail Making Test) in the fornix and cingulum, but diffusivity was not explored in this study. In another study, Serra et al. (2010) also observed a relationship between FA in the right anterior thalamic radiation and fornix and immediate memory recall (Rey Complex Figure Test) and between FA in the corpus callosum and delayed memory recall (15 Word Learning Test); again, diffusivity was not explored in this study.

Diffusion tensor imaging studies of cognition in healthy older adults are more numerous. These have also observed relationships between microstructural white matter, memory, and executive function, with executive function often demonstrating the greatest effect sizes [see Bennett and Madden (2014) for a review]. For example, a study by Cremers et al. (2016) explored the relationship between DTI metrics and tests of memory (15 Word Learning Test), processing and motor speed (Stroop Test; Purdue Pegboard Test), and executive function (Stroop Test; Letter Digit Substitution; Word Fluency) in healthy older adults. They found that white matter microstructure was related to processing speed and executive function, but not to memory performance in older adults.

The aim of the current study is to build upon our previous white matter microstructure findings (Mayo et al., 2017) and examine the relations between white matter microstructure and memory and executive function in persons with AD. We had three primary hypotheses. (1) Given our previous findings, we hypothesized that individuals with AD would have disrupted white matter integrity (lower FA and higher MD, AxD, RD) relative to healthy older adults. (2) The medial temporal regions involved in memory performance are thought to be one of the first regions affected in the progression of AD pathology (Braak and Braak, 1991; Hampel et al., 2014). We hypothesized that lower FA and higher diffusivity measures (MD, AxD, RD) would be associated with and lower memory test scores in regions of the medial temporal lobe in individuals with AD. (3) White matter structural changes in the frontal lobe are often observed in individuals with AD and some studies have found that these changes were related to executive dysfunction (Sjobeck et al., 2010). We hypothesized that lower FA and higher diffusivity measures (MD, AxD, RD) would be associated with lower executive function scores in the frontal regions of both groups.

## Materials and Methods

Data were obtained from the AD Neuroimaging Initiative 2 (ADNI2) database^1^. Led by Principal Investigator Dr. Michael W. Weiner, the ADNI was launched in 2003 with the goal of testing whether longitudinal brain imaging, biological markers, and neuropsychological assessment can be used together to measure the progression of AD. For more information, please see www.adni-info.org.

### Participants

Individuals with AD met NINCDS/ADRDA criteria for probable AD (McKhann et al., 1984), demonstrated abnormal memory function on the WMS Logical Memory II (≤8 for ≥16 years of education), scored between 20 and 26 on the MMSE, and scored either 0.5 (very mild) or 1.0 (mild) on the Clinical Dementia Rating.

Individuals from the ADNI database were included if both imaging (DTI) and neuropsychological data were available at the first time point (screening). Healthy older adults were free of subjective memory concerns, scored within the normal range on the WMS Logical Memory II (≥9 for ≥16 years of education), scored between 24 and 30 on the MMSE, and had a Clinical Dementia Rating of 0 (none). Full eligibility criteria are described in the ADNI2 Procedures Manual (Alzheimer’s Disease Neuroimaging Initiative, 2008).

Forty-nine individuals with AD and 48 matched healthy older adults were included in the current study. Participant demographics are presented in Table 1. All ADNI participants provided informed written consent approved by each study site’s Institutional Review Board. The current study was approved by the Human Research Ethics Boards at the University of Victoria, in British Columbia, Canada.

**Table 1:** Participant demographics and cognitive scores.

		Healthy older	AD vs. healthy
	AD	adults	older adults
Age	74.40 ± 8.52	72.92 ± 5.92	*p* = 0.320
Number of males	30	22	*p* = 0.131
Number of females	19	26	
Education	15.37 ± 2.87	16.36 ± 2.73	*p* = 0.079
ADNI-MEM	-0.92 ± 0.49	0.95 ± 0.62	*p* < 0.001
ADNI-EF	-0.85 ± 0.85	0.82 ± 0.79	*p* < 0.001


### MRI Image Acquisition

Imaging data were downloaded from the ADNI2 database. All participants had whole-brain MRI scans according to the ADNI protocol (Alzheimer’s Disease Neuroimaging Initiative, 2008). Specifically, images were acquired from 3T MRI scanners (GE Medical Systems). Axial diffusion weighted image data were acquired with a spin echo echo planar imaging sequence. Scan parameters were: acquisition matrix = 256 × 256, voxel size = 1.4 × 1.4 × 2.7 mm^3^, number of slices = 59. There were 46 images acquired for each scan: 41 diffusion-weighted images (*b* = 1000 s/mm^2^) and 5 non-diffusion-weighted images (*b* = 0 s/mm^2^). Repetition time varied across scanning sites, but was approximately 12,500–13,000 ms.

### Neuropsychological Data Acquisition

Neuropsychological data were downloaded from the ADNI2 database. All participants completed a battery of cognitive tests according to the ADNI protocol (Alzheimer’s Disease Neuroimaging Initiative, 2008) collected at the time of the MRI scans. Two composite scores derived from the ADNI neuropsychological battery were used in this study: (1) ADNI-MEM, a memory composite score and (2) ADNI-EF, an executive functioning composite score. Composite scores were chosen to minimize the effects of potential outlying performance on a single test item. ADNI-MEM was derived by confirmatory factor analysis using data from the Rey Auditory Verbal Learning Test (Trials 1–5, List B, Immediate Recall, Delayed Recall, and Recognition), ADAS-Cog (ADAS; Trials 1–3, Recall, Recognition Present, Recognition Absent), WMS Logical Memory I and II (Immediate and Delayed Recall), and Mini Mental State Exam (3 word recall). See Crane et al. (2012) for full details on psychometric development. ADNI-EF was derived by item response theory using data from the Wechsler Adult Intelligence Scale – Revised (Digit Symbol Substitution), Digit Span Backwards, Trails A and B, Category Fluency, and Clock Drawing [see Gibbons et al. (2012) for full details on psychometric development]. Although there are not many composite scores derived from traditional measures of executive functioning, the use of composite scores is well supported by evidence from meta-analytical examination of the structure of such complex ability (Karr et al., 2018).

### Data Analysis

Group-level demographic data (Table 1) was generated using R Studio (R Core Team, 2013). Imaging pre-processing and analyses were performed in Functional MRI of the Brain Software Library (FSL) (Analysis Group, FMRIB, Oxford, United Kingdom^2^; Smith et al., 2004). Diffusion images were corrected for head movement and eddy current distortions using Eddy Correct and non-brain tissue was removed using Brain Extraction Tool (Smith, 2002). Brain-extracted images were then visually inspected to confirm accurate removal of non-brain tissue. FA maps were then created using DTIfit and input into Tract-based Spatial Statistics (TBSS) to obtain a projection of all FA data onto a mean FA skeleton (Smith et al., 2006). First, all FA data were non-linearly aligned to common space (FMRIB58_FA), then the mean FA image was created and thresholded (FA > 0.2) to create a mean FA skeleton. Next, each participant’s FA data were projected onto the mean FA skeleton. TBSS was repeated for diffusivity measures (MD/AxD/RD). To do so, non-linear registration estimated from the FA images was applied to the diffusivity data and each participant’s MD/AxD/RD image was projected onto the mean FA skeleton, followed by voxel-wise statistical analysis. Voxel-wise statistical analysis of FA, MD, AxD, and RD in the white matter skeleton was performed using Randomize, FSL’s nonparametric permutation inference tool (Winkler et al., 2014). Group differences in FA, MD, AxD, and RD were evaluated. Relationships between DTI metrics with ADNI-MEM and ADNI-EF scores were evaluated in the AD and healthy older adult group separately, as well as after combining the two groups. Multiple comparisons were corrected for by using threshold-free cluster enhancement (*p* < 0.05). White matter regions were identified with Johns Hopkins University’s white matter atlas included in FSL (Wakana et al., 2007; Mori et al., 2008).

## Results

### FA, MD, AxD, and RD Differences Between AD and Healthy Older Adults

**FIGURE 1:**
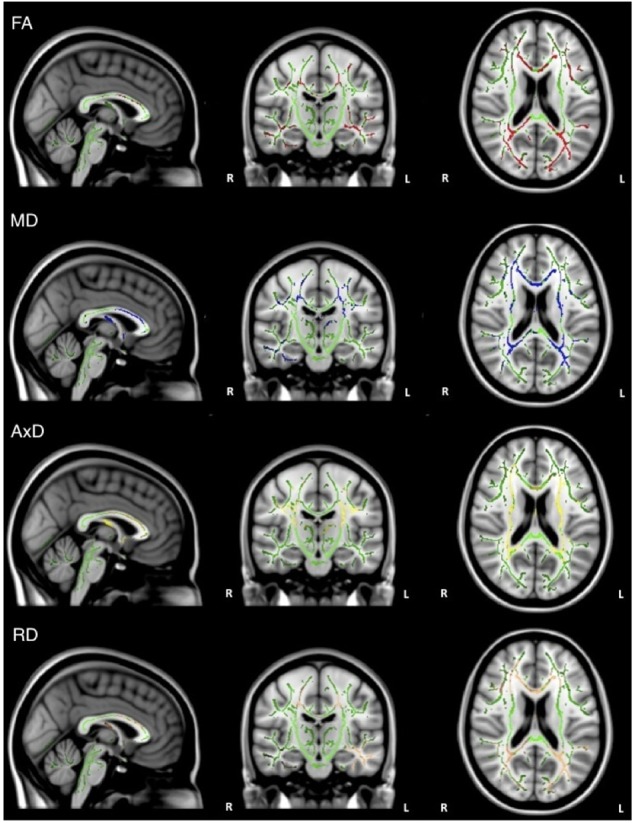
Pattern of low FA (red), high MD (blue), high AxD (yellow), and high RD (orange) overlaid on the white matter skeleton (green) in individuals with AD compared to healthy older adults (*p* < 0.05, corrected for multiple comparisons).

Consistent with our previous findings (Mayo et al., 2017), individuals with AD had significantly lower FA relative to healthy older adults in the corpus callosum, left internal capsule, corona radiata, posterior thalamic radiations, inferior longitudinal fasciculi, inferior fronto-occipital fasciculi, external capsule, cingulate gyri, right hippocampal cingulum, right fornix, superior longitudinal fasciculi, and tapetum. Individuals with AD had significantly higher MD relative to healthy older adults in the corpus callosum, internal capsule, corona radiata, left posterior thalamic radiation, left inferior longitudinal fasciculi, inferior fronto-occipital fasciculi, cingulate gyri, right hippocampal cingulum, fornix, superior longitudinal fasciculi, uncinate fasciculi, and tapetum. Additionally, individuals with AD had significantly higher AxD relative to healthy older adults in the corpus callosum, internal capsule, corona radiata, posterior thalamic radiations, external capsule, right fornix, superior longitudinal fasciculi, superior fronto-occipital fasciculi, and tapetum. Furthermore, individuals with AD had significantly higher RD relative to healthy older adults in the corpus callosum, left internal capsule, corona radiata, posterior thalamic radiations, inferior longitudinal fasciculi, inferior fronto-occipital fasciculi, external capsule, cingulate gyri, right hippocampal cingulum, left fornix, superior longitudinal fasciculi, left uncinate, and tapetum (Figure 1).

### Memory

**FIGURE 2:**
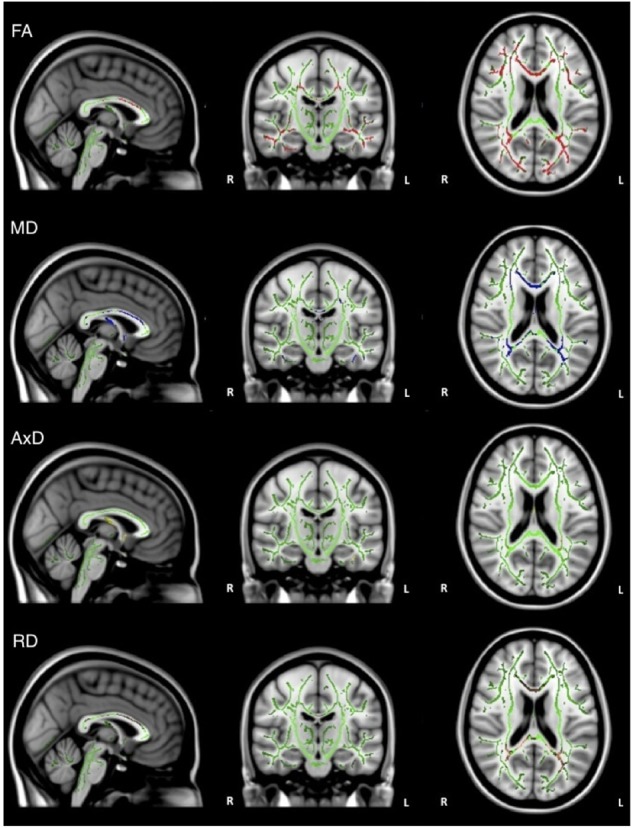
Regions where low ADNI-MEM memory scores are correlated with low FA (red), high MD (blue), high AxD (yellow), and high RD (orange) in the combined group of AD and healthy older adults (*p* < 0.05, corrected for multiple comparisons).

Overall, individuals with AD scored significantly lower than healthy older adults on memory test composite scores (Table 1). When the groups were combined (AD and healthy older adults), there was a significant positive relationship between FA and memory scores (ADNI-MEM) in widespread regions, including corpus callosum, internal capsule, corona radiata, posterior thalamic radiations, inferior longitudinal fasciculi, inferior fronto-occipital fasciculi, external capsule, left cingulate gyri, right hippocampal cingulum, fornix, superior longitudinal fasciculi, and tapetum. Similarly, there was a significant negative relationship between MD and memory scores in similar brain regions, including the corpus callosum, internal capsule, corona radiata, left posterior thalamic radiations, left inferior longitudinal fasciculus, cingulate gyri, fornix, superior longitudinal fasciculi, and tapetum. There was also a significant negative relationship between AxD and memory scores in the corpus callosum, left internal capsule, corona radiata, posterior thalamic radiations, left inferior longitudinal fasciculus, left inferior fronto-occipital fasciculus; fornix; and left superior longitudinal fasciculus, and tapetum. Similarly, there was a significant negative relationship between RD and memory scores in the corpus callosum, corona radiata, posterior thalamic radiations, superior longitudinal fasciculi, tapetum, and fornix (Figure 2). However, when the groups were examined separately, no significant relations between DTI metrics and memory scores were observed.

### Executive Function

**FIGURE 3:**
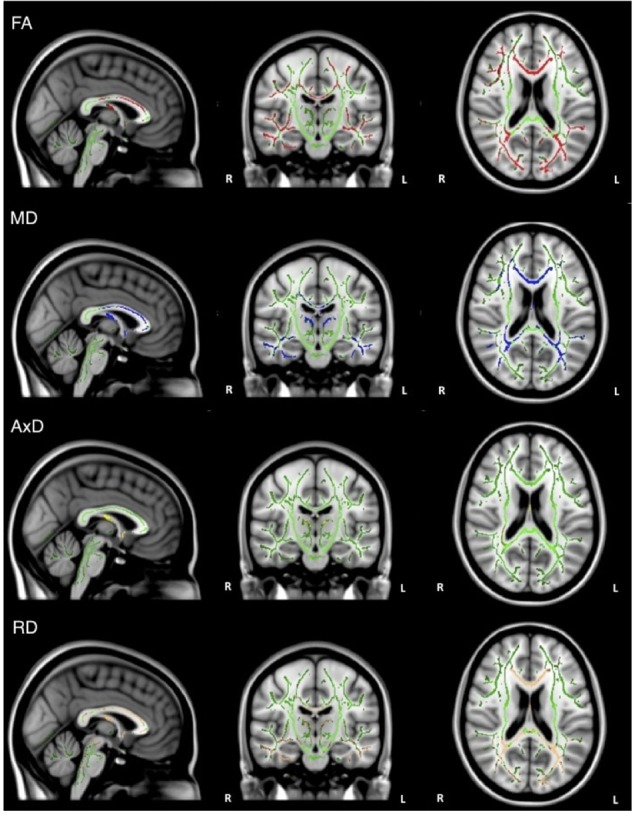
Regions where low ADNI-EF executive function scores are correlated with low FA (red), high MD (blue), high AxD (yellow), and high RD (orange) in the combined group of AD and healthy older adults (*p* < 0.05, corrected for multiple comparisons).

Overall, individuals with AD scored significantly lower than healthy older adults on composite scores for tests of executive function (Table 1). When the groups were combined (AD and healthy older adults), there was a significant positive relationship between FA and executive function scores (ADNI-EF) in widespread regions, including the corpus callosum, internal capsule, corona radiata, posterior thalamic radiation, inferior longitudinal fasciculi, inferior fronto-occipital fasciculi, external capsule, cingulate gyri, right hippocampal cingulum, left fornix, superior longitudinal fasciculi, uncinate fasciculi, and tapetum. There was also a significant negative relationship between MD and executive function scores in similar brain regions, including the corpus callosum, cerebral peduncles, internal capsule, corona radiata, posterior thalamic radiations, inferior longitudinal fasciculi, external capsule, cingulate gyri, right hippocampal cingulum, fornix, superior longitudinal fasciculi, uncinate fasciculi, and tapetum. Additionally, there was a significant negative relationship between AxD and executive function scores in the corpus callosum, right corona radiata, right posterior thalamic radiation, internal capsule, fornix, and right tapetum. Furthermore, there was a significant negative relationship between RD and executive function scores in the corpus callosum, cerebellar peduncles, fornix, corticospinal tract, medial lemniscus, cerebral peduncles, internal capsule, corona radiata, posterior thalamic radiations, inferior longitudinal fasciculi, inferior fronto-occipital fasciculi, external capsule, cingulate gyri, right hippocampal cingulum, fornix, superior longitudinal fasciculi, uncinate fasciculi, and tapetum (Figure 3).

**FIGURE 4:**
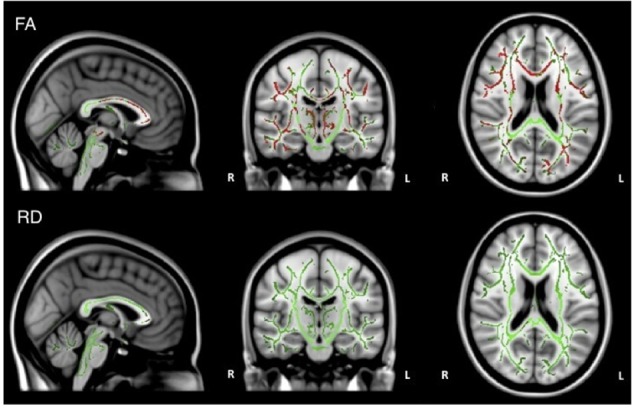
Regions where low ADNI-EF executive function scores are correlated with low FA (red) and high RD (orange) in healthy older adults (*p* < 0.05, corrected for multiple comparisons).

When the analysis was restricted to only individuals with AD, no significant relations between DTI metrics and executive function were observed. Healthy older adults did demonstrate a significant positive relationship between FA and executive function scores in the corpus callosum, corticospinal tract, medial lemniscus, cerebellar peduncle, internal capsule, superior corona radiata, posterior thalamic radiations, inferior longitudinal fasciculi, inferior fronto-occipital fasciculi, external capsule, cingulate gyri, right hippocampal cingulum, fornix, and right uncinate fasciculus. Additionally, there was a negative relationship between RD and executive function scores in the corpus callosum (Figure 4).

## Discussion

The current study aimed to examine (1) the white matter microstructure differences between individuals with AD and healthy older adults, (2) the relationship between white matter microstructure and memory, and (3) the relationship between white matter microstructure and executive function in these two groups. As predicted, we found lower FA and higher MD, AxD, and RD in the AD group compared to healthy older adults across widespread regions, including the hippocampal regions, as has been previously reported (Mayo et al., 2017). As expected, individuals with AD had poorer memory and executive function skills compared to healthy older adults. Below, we discuss in detail the findings related to the relationship between DTI metrics and memory and executive function.

### Memory

With respect to the relationship between DTI metrics and memory performance, we hypothesized that there would be a significant positive relationship between FA and memory scores and a significant negative relationship between diffusivity measures (MD/AxD/RD) and memory scores, suggesting that reduced white matter integrity is associated with poorer memory recall. Further, we hypothesized that the regions of white matter structural changes would include the medial temporal regions given that it is thought to be one of the regions first affected in AD (Braak and Braak, 1991).

When the groups were combined (AD and healthy older adults) we found significant relationships between all DTI metrics and composite memory scores (ADNI-MEM) across widespread regions of the brain, including the medial temporal regions as predicted. This is consistent with previous findings of DTI metrics and memory using combined groups including AD and healthy older adults (e.g., Bosch et al., 2012). However, when we examined the groups separately, we did not find any significant relationships between DTI metrics and memory test composite scores (ADNI-MEM) within either the AD or healthy older adult groups. The individuals with AD had significant white matter disruption and poorer memory scores, while the healthy older adults had relatively intact white matter and high memory scores. Significant relationships emerged between DTI metrics and cognitive scores when the groups (AD and healthy controls) were combined (but not when they were examined separately), likely given the slope created by the distribution of scores across the groups.

Previous studies have yielded mixed findings regarding the relationship between DTI metrics and memory. Some have found significant positive relationships between FA and memory in individuals with AD but have differed from ours in using either a single memory test rather than a composite measure (e.g., Serra et al., 2010), or in using a region of interest approach to the DTI analyses (e.g., Mielke et al., 2009). Neither study used diffusivity measures. Similarly, other studies have employed a region of interest approach to examine relationships between FA and memory in healthy older adults (e.g., Kantarci et al., 2011) and have reported significant relationships within specific regions (e.g., inferior longitudinal fasciculus, cingulum) but not others (fornix, superior longitudinal fasciculus, corticopontine tract, corpus callosum). Most such studies have focused on FA, and few have examined MD, despite evidence that MD may be more sensitive than FA in AD (Clerx et al., 2012; Nir et al., 2013). Thus, future research may consider including MD or other measures of diffusivity (such as AxD or RD), in addition to FA.

### Executive Function

With respect to the relationship between DTI metrics and executive function, we hypothesized that there would be a significant positive relationship between FA and executive function scores and a significant negative relationship between diffusivity measures (MD/AxD/RD) and executive function scores, suggesting that reduced white matter integrity is associated with poorer executive function. Further, we hypothesized that the regions of white matter structural changes would include frontal regions (Sjobeck et al., 2010).

When the groups were combined (AD and healthy older adults), there were significant relationships between all DTI metrics and executive function scores in widespread regions, including the frontal areas, as predicted. Nir et al. (2013) also examined these relationships using ADNI’s ADNI-EF composite scores (but in a combined cohort of AD and adults with mild cognitive impairment) and found a similar relationship between ADNI-EF scores and FA/MD.

When examined separately, we did not find significant relationships between DTI metrics and executive function composite scores in AD. Again, previous findings on this issue are mixed. For example, Serra et al. (2010) found a significant relationship between FA in the right anterior thalamic radiation and fornix, and performance on a test that has some executive function requirements (i.e., Rey Complex Figure Test) when they examined a combined cohort that combined AD and adults with mild cognitive impairment. However, Bosch et al. (2012) did not observe a significant relationship between FA or diffusivity measures and a different composite measure of executive function (Digit backwards, Symbol search, Wechsler Adult Intelligence Scales similarities, and Controlled Oral Word Reading Test) in their study of an AD only sample.

Our finding of significant relationships between FA and executive function in healthy older adults is consistent with previous literature (e.g., Grieve et al., 2007; Kennedy and Raz, 2009; Cremers et al., 2016). Given this overall pattern, is it possible that the significant relationships between FA and executive function observed in the combined group (AD and healthy older adults) are largely driven by the healthy older adults.

### Limitations and Future Directions

The current study had a number of limitations. We opted for a whole-brain TBSS approach, rather than an ROI approach, to best characterize all of the regions that may be related to memory or executive function. The whole-brain TBSS approach has an increased risk of Type I errors (false positives) given the multiple comparisons, relative to a region of interest approach. We employed threshold-free cluster enhancement to reduce the Type I error risk, however, but it is important to consider that some of the significant voxels may represent Type I error.

We relied on ADNI-derived composite measures (ADNI-MEM and ADNI-EF) to explore the relationship between DTI metrics and cognitive functioning. These composite measures were chosen to minimize the effects of a potential outlier on a single test; however, it is possible that the ADNI-MEM and ADNI-EF composite scores are not the most sensitive measures of “memory” and “executive” functions, in terms of reflecting white matter microstructure. Future research may benefit from exploring the relationship between white matter integrity and cognitive function using other cognitive test scores, including individual cognitive tests and/or other cognitive composites.

Additionally, the sample size is limited, as is common in neuroimaging research, which may have limited the statistical power of our separate group analyses. Data collection for ADNI3 is on-going. Future research may benefit from including additional participants, across multiple time points, to better characterize the relationship between white matter integrity and cognitive function. As the ADNI AD group represents the earliest stages of AD, it is possible that differences may emerge over time. Furthermore, future research should look to include other groups at high risk of developing AD (e.g., mild cognitive impairment, subjective cognitive decline), including those who convert to AD over the course of the study, as further longitudinal data become available through ADNI. Future research may also wish to explore longitudinal gray matter and white matter changes in combination (e.g., Delli Pizzi et al., 2015), including tools designed to assess DTI metrics in subcortical gray matter, in order to help evaluate whether white matter may change independently and prior to changes in gray matter.

## Conclusion

White matter microstructure, memory, and executive function are disrupted in AD. However, the current findings suggest that there may not be a linear relationship between the degree of white matter disruption and level of cognitive function (memory and executive function) in AD. Given the diversity in research findings to date, future research should continue to explore the relationships between DTI metrics and cognitive function to determine whether DTI holds potential to measure clinical progression of AD and/or treatment efficacy.

## Data Availability Statement

The raw data supporting the conclusions of this manuscript will be made available by the authors, without undue reservation, to any qualified researcher. Please email requests to: gawryluk@uvic.ca. Publicly available datasets were analyzed in this study. This data can be found at: http://adni.loni.usc.edu.

## Author Contributions

CM and JG performed the literature search and writing of the first draft. CM, MG-B, and JG were involved in data analysis. CM, MG-B, EM, LR, JF and JG performed writing and editing subsequent versions of manuscript and approving submission.

## Conflict of Interest Statement

The authors declare that the research was conducted in the absence of any commercial or financial relationships that could be construed as a potential conflict of interest.

## Footnotes

1 http://adni.loni.usc.edu

2 http://fsl.fmrib.ox.ac.uk
